# Epidemiological overview of *Cryptosporidium* infection in *Capra hircus* from southern Khyber Pakhtunkhwa, Pakistan

**DOI:** 10.5455/javar.2026.m1062

**Published:** 2026-06-22

**Authors:** Naimat Ullah Khan, Farhad Badshah, Tahir Usman, Muhammad Salman Khan, Jamil Ur Rehman, Riaz Amin, Eliana Ibáñez-Arancibia, Warda Naz, Hazrat Ali, Mian Saeed Sarwar, Patricio R. De los Ríos Escalante, Mourad Ben Said, Hanène Belkahia

**Affiliations:** 1 College of Veterinary Sciences and Animal Husbandry, Abdul Wali Khan University Mardan, Mardan 23200, Khyber Pakhtunkhwa, Pakistan; 2 Department of Zoology, Abdul Wali Khan University Mardan, Mardan 23200, Khyber Pakhtunkhwa, Pakistan; 3 State Key Laboratory of Animal Biotech Breeding, Institute of Animal Science, Chinese Academy of Agricultural Sciences, Beijing 100193, China; 4 Shenzhen Branch, Guangdong Laboratory of Lingnan Modern Agriculture, Key Laboratory of Livestock and Poultry Multi-Omics of MARA, Agricultural Genomics Institute at Shenzhen, Chinese Academy of Agricultural Sciences, Shenzhen 518000, China; 5 Department of Zoology, University of Science and Technology Bannu, Bannu 28100, Khyber Pakhtunkhwa, Pakistan; 6 Department of Zoology, Kohat University of Science and Technology, Kohat 26000, Khyber Pakhtunkhwa, Pakistan; 7 Department of Zoology, University of Lakki Marwat, Lakki Marwat 28420, Khyber Pakhtunkhwa, Pakistan; 8 PhD Program in Sciences Mentioning Applied Molecular and Cell Biology, La Frontera University, Temuco, Chile; 9 Laboratory of Engineering, Biotechnology, and Applied Biochemistry (LIBBA), Department of Chemical Engineering, Faculty of Engineering and Science, La Frontera University, Temuco, Chile; 10 Departamento de Ciencias Biologicas y Quimicas, Facultad de Recursos Naturales, Universidad Catolica de Temuco, Chile; 11 Department of Zoology, Hazara University, Mansehra 21300, Khyber Pakhtunkhwa, Pakistan; 12 Nucleo de Estudios Ambientales, Facultad de Recursos Naturales, Universidad Catolica de Temuco, Chile; 13 Department of Basic Sciences, Higher Institute of Biotechnology of Sidi Thabet, University of Manouba, Manouba 2010, Tunisia; 14 Laboratory of Microbiology, National School of Veterinary Medicine of Sidi Thabet, University of Manouba, Manouba 2010, Tunisia

**Keywords:** Cryptosporidiosis, *Cryptosporidium* infection, microscopic examination, prevalence, Pakistan, risk factors

## Abstract

**Objectives:** *Cryptosporidium* is a protozoan parasite that commonly infects livestock, including *Capra hircus*, causing diarrhea, dehydration, and sometimes mortality. These infections lead to substantial economic losses in the farming industry. This study aimed to determine the prevalence and associated risk factors, such as region, season, age, and sex, of *Cryptosporidium* infection in *C. hircus* in southern Khyber Pakhtunkhwa, Pakistan.

**Materials and Methods:** A total of 360 fecal samples were collected from *C. hircus* from the Kohat, Bannu, and Lakki Marwat Districts of Khyber Pakhtunkhwa, Pakistan. Fecal smears were stained using the modified Ziehl-Neelsen method, and statistical analysis, including chi-square tests, was employed to assess the association between *Cryptosporidium* infection prevalence and various factors.

**Results:** The overall *Cryptosporidium* infection prevalence was 20.55% (74/360). District-wise analysis revealed the highest prevalence in Kohat (23.33%), followed by Bannu (20%) and Lakki Marwat (18.33%). Monthly examination identified August as the peak prevalence month (36.67%), displaying non-significant monthly variations (*p* = 0.075). Seasonal disparity indicated a significantly higher prevalence in the summer (30%) compared to other seasons (*p* = 0.008). Age-wise prevalence demonstrated susceptibility in kids aged ≤ 15 days (≤ 33.93%), with significant overall age-based differences (*p* = 0.001). Female kids exhibited a slightly higher prevalence at 22.22% compared to males (19.19%), with no significant gender-based difference (*p* = 0.479).

**Conclusions:** This study highlights the influence of geographic, seasonal, and demographic factors on *Cryptosporidium* prevalence in goat populations. The findings emphasize the importance of implementing targeted preventive measures. Recommended strategies include farmer education programs, seasonal deworming schedules, early screening for infection, and strict hygiene and water sanitation practices to reduce transmission risks effectively.

## 1. Introduction

*Cryptosporidium* is an intracellular, obligate protozoan parasite that affects the digestive system and has both medical and veterinary significance. It is responsible for infections in a wide range of vertebrates [1]. Goat farming plays a vital role in Pakistan’s rural economy, providing income, meat, and milk to millions of smallholder farmers, especially in resource-limited settings. This livestock sector is critical for food security and poverty alleviation. However, infectious diseases such as cryptosporidiosis threaten goat health and productivity, with economic consequences for farmers. Moreover, *Cryptosporidium* infections in goats pose a public health concern because zoonotic transmission to humans is well documented, especially in regions with close human-animal contact. Although data about *Cryptosporidium* prevalence in goats and humans in Pakistan exist, information remains limited, underscoring the need for epidemiological studies to inform control strategies [1].

*Cryptosporidium* species are obligatory intracellular parasites affecting both humans and animals, with documented cases on every continent except Antarctica. Globally, 24 foodborne diseases have been identified, and cryptosporidiosis stands out as the fifth most significant among them [2]. This disease has distinct clinical characteristics and generally has a favorable prognosis in immunocompetent individuals but posing a greater risk in those who are immunodeficient [3]. It manifests primarily as gastrointestinal issues such as enteritis, diarrhea, dysentery, and abdominal pain, cryptosporidiosis exhibits fewer clinical signs related to respiratory, pancreatic, and hepatic disorders. Cryptosporidiosis is one of the most common zoonotic diseases caused by various species of *Cryptosporidium*, such as *C. hominis, C. parvum*, and *C. ubiquitum* [4].

The genus *Cryptosporidium* is composed of multiple species that infect a broad range of animals, contributing to its zoonotic potential. Banda [5] documented the prevalence of *Cryptosporidium* in diarrheic children in Zambia, emphasizing its zoonotic transmission in regions with close human-animal interaction. Similarly, Cheng et al. [6] conducted a study on the prevalence of *Cryptosporidium* spp. in sheep and goats in Jiangsu, China, identifying *C. xiaoi* as a key species, highlighting the zoonotic risks associated with these livestock. Mahen et al. [7] investigated the prevalence and risk factors of *Cryptosporidium* spp. in buffaloes in Bangladesh, expanding the known animal reservoirs of the parasite in South Asia. The study highlighted significant infection rates in younger and diarrheic animals, emphasizing the necessity of targeted control measures.

Further complicating the understanding of its transmission, Akinnubi [8] presented evidence of respiratory transmission via inhalation, suggesting that *Cryptosporidium* may spread through routes beyond fecal-oral transmission. The study conducted by Ryan et al. [9] provided an update on zoonotic *Cryptosporidium* species and genotypes in humans. They highlighted the importance of DNA-based detection and genetic typing methods to identify species capable of infecting humans. Their work emphasized the need for more studies in regions with high zoonotic transmission risks and the application of advanced genetic tools to further understand the zoonotic transmission of this important parasite. Olonisakin and Olusi [10] investigated the prevalence of *Cryptosporidium* spp. in free-range and poultry birds in Akure South LGA, Ondo State, Nigeria. Their study found an overall prevalence of 11.9% and demonstrated that free-range birds had a higher infection rate (13.2%) than poultry birds (10.9%), confirming that birds, particularly in semi-intensive farming systems, can contribute to the zoonotic transmission of *Cryptosporidium*. Díaz et al. [11] provided a broader perspective on cryptosporidiosis as a global public health concern, particularly in regions with poor sanitation. Lastly, Ryan and Power [12], as well as Ng et al. [13], contributed to understanding the epidemiology of *Cryptosporidium* in Australian wildlife and livestock, revealing the zoonotic potential in diverse animal hosts and emphasizing the need for preventive measures in agricultural settings. Recently, a few species of *Cryptosporidium* have been identified in goats, such as *Cryptosporidium parvum, C. ubiquitum, C. hominis,* and *C. xiaoi* [14]. Currently, 47 species and 120 genotypes of *Cryptosporidium* have yet to be fully classified [15–17]. Globally, *Cryptosporidium* species are considered a primary cause of parasitic diarrhea [18, 19].

*Cryptosporidium* and *Giardia* species are implicated in the occurrence of parasitic diarrhea in humans, with prevalence rates ranging from 1% to 3% in advanced countries and 4% to 17% in less developed countries [19]. In African countries, cryptosporidiosis is one of the major causes of morbidity and mortality in children, and it is claimed that transmission is mainly anthroponotic. This may be related to close contact between humans and animals; however, human-to-human transmission has also been reported [20]. Developed countries have witnessed significant outbreaks of *Cryptosporidium* infection transmitted through food and water, primarily attributed to inadequate hygiene practices and the resilience of these parasites to conventional disinfectants such as chlorine, as well as the increasing zoonotic potential of the parasite. Recent reviews emphasize the importance of a One Health approach to understanding and managing these outbreaks, highlighting the need for better surveillance, preventative measures, and management policies [2, 21]. *Cryptosporidium* infection tends to be prominently reported in children under the age of five, with malnourished children exhibiting a higher susceptibility compared to their healthier counterparts [6, 22].

In 1981, the first recorded case of cryptosporidiosis in Australia involved a two-week-old *C. hircus* suffering from diarrhea [23]. *Cryptosporidium* infection holds significant economic implications in goats, leading to substantial losses and posing health risks to humans due to its zoonotic nature. A study conducted over a four-week period revealed a notable 2 kg body weight difference between healthy and *Cryptosporidium*-infected *C. hircus* [24]. The prevalence of *Cryptosporidium* infection in *C. hircus* ranges from 5% to 35%, as reported by various researchers [25]. Additionally, *C. parvum* has been identified in goats in several countries, including Zambia, Australia, Cyprus, and Belgium, as summarized by Ali et al. [26].

*Cryptosporidium* is a global contributor to cryptosporidiosis outbreaks, serving as the primary cause of enteric infections in humans, while animals, particularly young small and large ruminant species, act as reservoirs [20]. Previous reports indicate a *Cryptosporidium* prevalence ranging from 5% to 77% worldwide [27, 28]. In the Canary Islands (Spain), a 23.3% and 2.5% prevalence of *Cryptosporidium* infection has been documented in *C. hircus* and goat kids [29]. Molecular data are limited and available concerning goat cryptosporidiosis, with only a few genotypes and species identified, such as *C. parvum* and *C. hominis*. Two early studies found *Cryptosporidium* genotypes in young goats, specifically *C. xiaoi* and *C. parvum* [30, 31].

Given that *C. parvum* and *C. hominis,* both responsible for human cryptosporidiosis, are also found in goats, the significance of goat cryptosporidiosis as a zoonosis cannot be overlooked. In Pakistan, limited data exist regarding the prevalence of cryptosporidiosis in *C. hircus* and its potential implications for human health, whereas the prevalence in large ruminants has received slightly more attention with a few available publications. In this study, our objective is to provide information on the prevalence, associated risk factors, and the potential public health risk posed by *Cryptosporidium* infection in *C. hircus* due to zoonotic transmission.

## 2. Materials and Methods

### 2.1. Ethical approval

This research study was ethically approved (Approval No. UVAS-2017-PHD-CM-004) by the Ethical Approval Committee of the University of Veterinary and Animal Sciences, Lahore, Pakistan.

### 2.2. Study area

**Figure 1.**
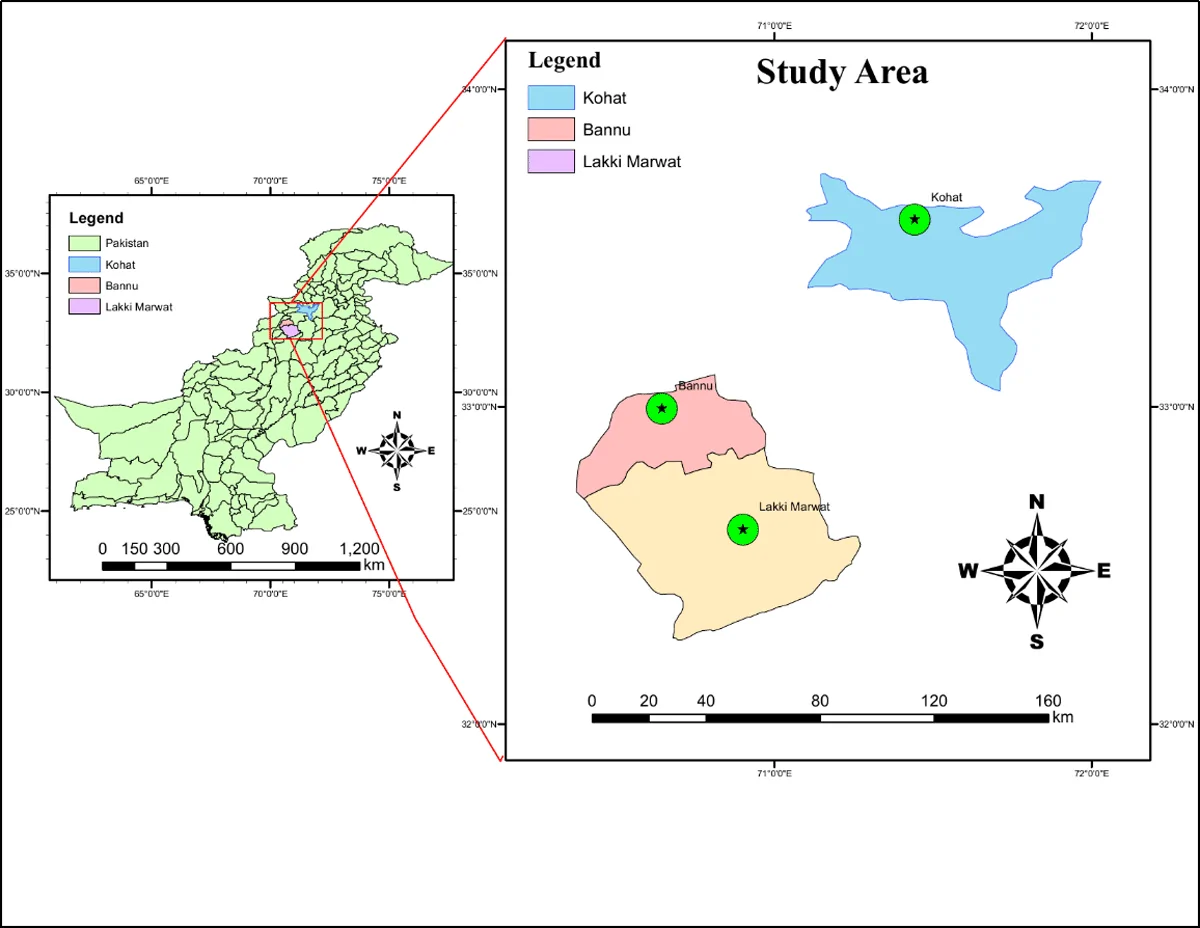
Study area map.

Fieldwork was conducted in three districts of southern Khyber Pakhtunkhwa, Pakistan: Bannu (32.99°N, 70.61°E; elevation 371 m), Lakki Marwat (32°36′27″N, 70°54′45″E), and Kohat (32°47′–33°53′N, 70°34′–72°17′E) (Figure 1). These districts were purposively selected due to their dense populations of small ruminants, managed primarily under traditional and semi-intensive systems that encourage close animal contact, communal grazing, and often insufficient hygiene, a condition recognized as facilitating *Cryptosporidium* transmission. The predominant vegetation in these regions consists of xerophytic shrubs and sparse grasses, providing limited grazing resources and promoting animal congregation at communal water points, further elevating infection risk. The area is characterized by semi-arid to arid climatic conditions, with pronounced seasonal variability. Meteorological data for the study period showed mean monthly temperatures ranging from 5°C in winter to peaks of 45°C during summer and relative humidity oscillating between 25% and 65%. Annual rainfall averages 300–600 mm, with sporadic heavy downpours concentrated in the monsoon months (July–September). Such climatic features likely influence the environmental persistence and transmission dynamics of *Cryptosporidium* oocysts: high temperatures may reduce oocyst viability, whereas rainfall events increase surface runoff, potentially contaminating water sources and grazing areas.

Ecologically, the presence of temporary water ponds and frequent use of stream water for animal husbandry were observed, together with widespread use of communal corrals. The movement of livestock between villages and use of open grazing lands create frequent contact between herds from different management units, amplifying the risk of cross-infection. The local economy is heavily reliant on livestock, underlining the veterinary and public health significance of zoonotic pathogens such as *Cryptosporidium*. All biological samples collected were processed and analyzed at the Central Diagnostic Laboratory, UVAS, under standardized conditions.

### 2.3. Experimental sampling strategy

Fecal samples were collected directly per rectum or from freshly passed feces of asymptomatic *C. hircus* using a convenient sampling method over a one-year period. Convenience sampling was employed due to logistical and operational constraints, including limited access to comprehensive farm records and the scattered nature of smallholder goat herds in the study area. Cotton swabs were employed to collect the samples, and relevant information was recorded on a questionnaire, including the owner’s name, address, contact details, animal characteristics (sex and age), and the prevailing season (winter, autumn, spring, and summer). A total of 360 samples were collected over 12 months from three distinct districts and subjected to Leica DM1000 light microscope (Leica Microsystems, Germany) examination following modified Ziehl-Neelsen acid-fast staining. Permission from the owners was obtained before sample collection, and, in return, free veterinary care and services, such as treatment, vaccination, and extension messages, were provided without any associated fees or charges for medicine. This study adhered to ethical guidelines and practices throughout the sampling process.

### 2.4. Sample collection and handling protocol

Fecal samples, approximately 2–3 gm each, were directly obtained from the rectum of *C. hircus* using cotton swabs or freshly defecated feces. Subsequently, these samples were preserved in 10% formalin at 1:3. Samples were preserved in sterile, clean, moisture-resistant, labeled, and disposable wide-mouthed plastic bottles, ensuring no contamination with urine. The samples were then refrigerated at 4°C until analysis within one or two weeks. Analysis of the fecal samples was conducted using the Faust modified centrifuge-flotation technique, following the procedure outlined by Khan et al. [32]. To create a uniform solution, 3 gm of fecal material was dissolved in distilled water, and a homogenized solution was prepared after centrifugation at 1500 rpm. After centrifugation, the supernatant was removed, and the sediment was resuspended in a flotation solution, ZnSO₄ (44%). The suspension was centrifuged again at 1500 rpm for 1 min. Finally, the sediment was examined under a microscope, and the supernatant was discarded. *Cryptosporidium* oocysts were stained using the modified Ziehl-Neelsen (MZN) staining technique [33], and positive samples were isolated to confirm the presence of *Cryptosporidium* oocysts (Sigma-Aldrich, Germany).

### 2.5. Fecal smear staining for Cryptosporidium oocyst identification

A thin fecal smear was prepared and fixed in methanol for 2–3 min. The smear was then stained with strong carbolfuchsin for 15–20 min, followed by rinsing with tap water. Decolorization was performed using 1% acid alcohol for 2 min and then rinsed again. Methylene blue was applied as a counterstain for 1 min, followed by a final rinse and air drying. All slides were examined under a Leica DM1000 light microscope (Leica Microsystems, Germany) at 400x magnification with oil immersion, following the procedure described by Ferreira et al. [34].

*Cryptosporidium* oocysts appeared as bright red, spherical to ovoid structures against a blue-green background in the modified Ziehl-Neelsen (MZN) stained fecal smears. Identification was based on established morphological criteria, including the presence of a characteristic double-layered oocyst wall, a size ranging between 4 and 6 μm, and a centrally located sporozoite visible within the oocyst, which appears as a refractile body under high magnification. These features were used to distinguish *Cryptosporidium* oocysts from other acid-fast organisms or artifacts.

A sample was considered positive when at least one clearly identifiable *Cryptosporidium* oocyst met these diagnostic criteria. The prevalence rate (%) was calculated using the formula:


\[
{\rm Prevalence \left(\%\right) = \left(\right. Number\, of\, infected\, individuals\, at\, a\, given\, time\, Number\, of\, individuals\, at\, risk\, at\, that\, time \left.\right) \times 100}
\]

For microscopy, a single slide per fecal sample was prepared and examined. An animal was deemed positive if at least one oocyst was detected on that slide. It is important to note that the modified Ziehl-Neelsen staining technique, while widely used for field-level diagnosis due to its relative simplicity and cost-effectiveness, has inherent limitations in terms of diagnostic performance. Reported sensitivity ranges between 70 and 80%, and specificity exceeds 90%, depending on the quality of sample preparation and examiner expertise [35]. Therefore, false negatives can occur, particularly in samples with low oocyst shedding, and the method is not suitable for differentiating *Cryptosporidium* species. These limitations imply that prevalence figures reported in this study may underestimate the true infection rates. Complementary molecular diagnostics could improve detection accuracy but were beyond the scope of the present study.

### 2.6. Statistical analysis

A chi-square (χ²) test was employed using SPSS Statistics 26 to assess the association between infection occurrence and various risk factors, including age, sex, geographical area, and season. A significance level of *p* < 0.05 was applied, considering values below this threshold as statistically significant.

## 3. Results

### 3.1. Microscopic examination of Cryptosporidium oocysts

**Figure 2.**
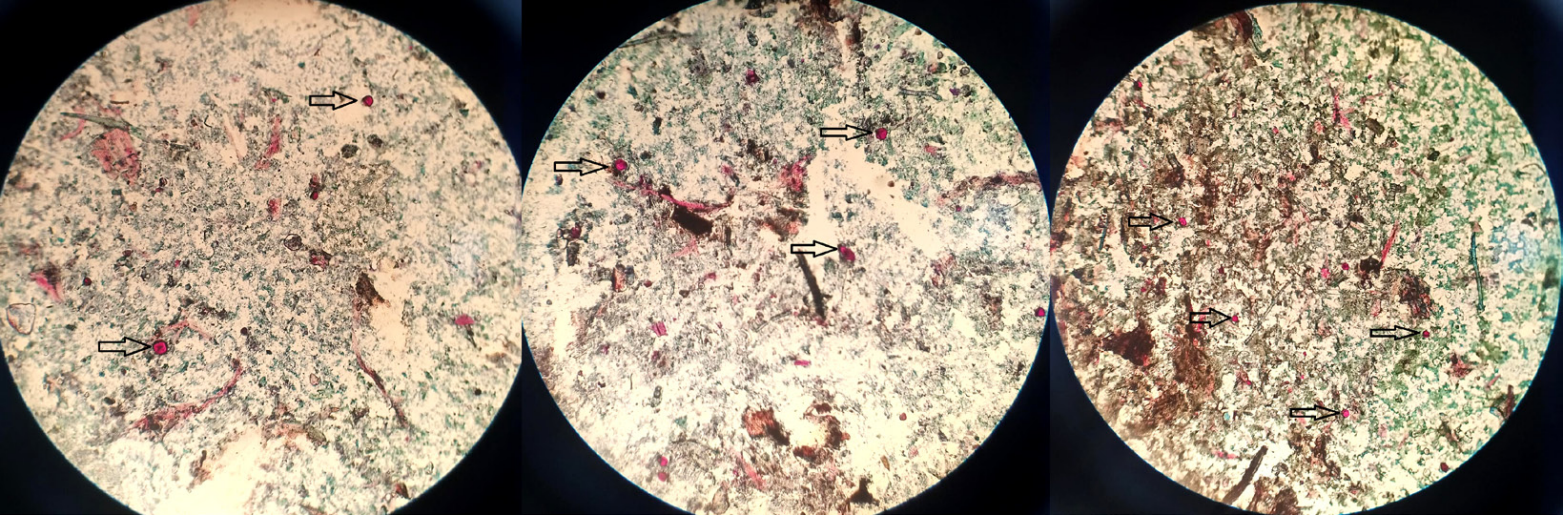
Microscopic observation of *Cryptosporidium* oocysts stained with the MZN method at 400x magnification.

Microscopic analysis revealed *Cryptosporidium* oocysts in fecal samples from *C. hircus*, characterized by their spherical or ovoid shapes, double-layered walls, and centrally located sporozoites (Figure 2).

### 3.2. Prevalence of Cryptosporidiosis in C. hircus

The overall prevalence of cryptosporidiosis among *C. hircus* was estimated at 20.55%. Infection rates varied across districts, with the highest prevalence in Kohat at 23.33%, followed by Bannu at 20%. Lakki Marwat recorded the lowest prevalence at 18.33% (Table 1).

**Table 1.** Monthly and seasonal prevalence of cryptosporidiosis in *C. hircus* in three selected districts of southern Khyber Pakhtunkhwa, Pakistan.

Factors	Classes	Bannu		Lakki Marwat		Kohat		Total	
		Infected/total (Rate, % ± C.I.^1^)	*p* value	Infected/total (Rate, % ± C.I.^1^)	*p* value	Infected/total (Rate, % ± C.I.^1^)	*p* value	Infected/total (Rate, % ± C.I.^1^)	*p* value
Months	December	1/10 (10 ± 0.19)	0.530	2/10 (20 ± 0.25)	0.285	2/10 (20 ± 0.25)	0.492	5/30 (16.67 ± 0.13)	0.075
	January	0/10 (0.00)		0/10 (0.00)		2/10 (20 ± 0.25)		2/30 (6.67 ± 0.09)	
	February	2/10 (20 ± 0.25)		0/10 (0.00)		0/10 (0.00)		2/30 (6.67 ± 0.09)	
	March	1/10 (10 ± 0.19)		3/10 (30 ± 0.28)		1/10 (10 ± 0.19)		5/30 (16.67 ± 0.13)	
	April	3/10 (30 ± 0.28)		1/10 (10 ± 0.19)		3/10 (30 ± 0.28)		7/30 (23.33 ± 0.15)	
	May	4/10 (40 ± 0.30)		2/10 (20 ± 0.25)		3/10 (30 ± 0.28)		9/30 (30.00 ± 0.16)	
	June	2/10 (20 ± 0.25)		3/10 (30 ± 0.28)		3/10 (30 ± 0.28)		8/30 (26.67 ± 0.16)	
	July	3/10 (30 ± 0.28)		4/10 (40 ± 0.30)		1/10 (10 ± 0.19)		8/30 (26.67 ± 0.16)	
	August	3/10 (30 ± 0.28)		3/10 (30 ± 0.28)		5/10 (50 ± 0.31)		11/30 (36.67 ± 0.17)	
	September	1/10 (10 ± 0.19)		2/10 (20 ± 0.25)		3/10 (30 ± 0.28)		6/30 (20 ± 0.14)	
	October	3/10 (30 ± 0.28)		2/10 (20 ± 0.25)		3/10 (30 ± 0.28)		8/30 (26.67 ± 0.16)	
	November	1/10 (10 ± 0.19)		0/10 (0.00)		2/10 (20 ± 0.25)		3/30 (10 ± 0.11)	
Seasons	Winter	3/30 (10 ± 0.11)	0.289	2/30 (6.67 ± 0.09)	0.050	4/30 (13.33 ± 0.12)	0.455	9/90 (10% ± 0.05)	0.008*
	Spring	8/30 (26.67 ± 0.16)		6/30 (20 ± 0.14)		7/30 (23.33 ± 0.15)		21/90 (23.33 ± 0.09)	
	Summer	8/30 (26.67 ± 0.16)		10/30 (33.33 ± 0.17)		9/30 (30 ± 0.16)		27/90 (30 ± 0.09)	
	Autumn	5/30 (16.67 ± 0.13)		4/30 (13.33 ± 0.12)		8/30 (26.67 ± 0.16)		17/90 (18.89 ± 0.08)	
Total		24/120 (20 ± 0.07)		22/120 (18.33 ± 0.07)		28/120 (23.33 ± 0.08)		74/360 (20.55 ± 0.04)	

Abbreviations: ^1^:C.I.: 95% confidence interval, *: statistically significant, *p* < 0.05.Note: The seasons are defined as follows: Winter (December, January, and February), Spring (March, April, and May), Summer (June, July, and August), and Autumn (September, October, and November).

### 3.3. Monthly variation in cryptosporidiosis prevalence

August showed the highest monthly prevalence at 36.67%, followed by May at 30%. Other notable rates included June, July, and October at 26.67%, April at 23.33%, and September at 20%. March and December each reported a prevalence of 16.67%, while November’s rate was 10%. The lowest prevalence occurred in January and February at 6.67%. Statistical analysis indicated a non-significant difference (*p* = 0.075) in prevalence rates across months (Table 1).

### 3.4. Seasonal variation in cryptosporidiosis prevalence

The summer season had the highest prevalence at 30%, followed by spring and autumn at 23.33% and 18.89%, respectively. Conversely, winter exhibited the lowest prevalence at 10%. Statistical analysis confirmed a significant difference (*p* = 0.008) in infection rates across seasons (Table 1).

### 3.5. Prevalence of cryptosporidiosis by age and gender

The highest prevalence was observed in *C. hircus* aged ≤15 days at 33.93%, followed by 17.69% in the 16–30-day age group. The lowest prevalence, at 11.01%, was recorded among kids aged 31–60 days. Statistical analysis highlighted a significant difference among the age groups (*p* < 0.001) (Table 2). Regarding gender, female kids showed a slightly higher prevalence (22.22%) compared to males (19.19%), but the difference was not statistically significant (*p* = 0.479) (Table 2).

**Table 2.** Prevalence of cryptosporidiosis according to age and sex of *C. hircus* located in three selected districts of southern Khyber Pakhtunkhwa, Pakistan.

Factors	Classes	Bannu		Lakki Marwat		Kohat			Total
		Infected/total (Rate, % ± C.I.^1^)	*p* value	Infected/total (Rate, % ± C.I.^1^)	*p* value	Infected/total (Rate, % ± C.I.^1^)	*p* value	Infected/total (Rate, % ± C.I.^1^)	*p* value
Age	≤ 15 days	12/36 (33.33 ± 0.15)	0.038*	10/32 (31.25 ± 0.16)	0.068	16/44 (36.36 ± 0.14)	0.028*	38/112 (33.93 ± 0.09)	< 0.001*
	16–30 days	9/52 (17.31 ± 0.10)		7/42 (16.67 ± 0.11)		7/36 (19.44 ± 0.13)		23/130 (17.69 ± 0.06)	
	31–60 days	3/32 (9.38 ± 0.10)		5/46 (10.87 ± 0.09)		5/40 (12.50 ± 0.10)		13/118 (11.01 ± 0.06)	
Sex	Male	12/64 (18.75 ± 0.10)	0.715	10/64 (15.62 ± 0.09)	0.414	16/70 (22.86 ± 0.10)	0.884	38/198 (19.19 ± 0.05)	0.479
	Female	12/56 (21.43 ± 0.11)		12/56 (21.43 ± 0.11)		12/50 (24.00 ± 0.12)		36/162 (22.22 ± 0.06)	
Total		24/120 (20 ± 0.07)		22/120 (18.33 ± 0.07)		28/120 (23.33 ± 0.08)		74/360 (20.55 ± 0.04)	

Abbreviations: ^1^: C.I.: 95% confidence interval, *: statistically significant, *p* < 0.05.

## 4. Discussion

The occurrence of *Cryptosporidium* infection in *C. hircus* has garnered attention globally, with numerous researchers documenting its prevalence in various countries. The rates of prevalence and incidence exhibit considerable variation, influenced by factors such as geographical distribution and sample size. This study contributes a pioneering report on cryptosporidiosis prevalence in *C. hircus* in southern Khyber Pakhtunkhwa, Pakistan, adding to the existing body of knowledge. Earlier investigations by different researchers have reported prevalence rates ranging from 5% to 35%; these findings emphasize the geographic variability of cryptosporidiosis [25] and support the role of goats, cattle, and sheep as potential reservoirs for zoonotic transmission of *Cryptosporidium* to humans [36]. Furthermore, the zoonotic potential of *C. parvum*, a species of concern, has been documented in small ruminants in countries like Zambia, Belgium, Australia, and Cyprus [31]. This underscores the global significance of understanding and addressing *Cryptosporidium* infection in goat populations.

This study documented an overall *Cryptosporidium* infection prevalence of 20.55% among *C. hircus* across three distinct districts, with notable spatial variability: the highest infection rate was observed in District Kohat (23.33%), followed by District Bannu (20%), and the lowest in District Lakki Marwat (18.33%). The observed district-wise differences likely reflect the interplay of multiple environmental, ecological, and management factors influencing parasite transmission dynamics. For instance, District Kohat’s relatively higher humidity and moderate temperatures create a more favorable microclimate for *Cryptosporidium* oocyst survival and infectivity, contrasting with the drier, hotter conditions of Lakki Marwat that may accelerate oocyst desiccation and reduce their environmental persistence.

Moreover, Kohat’s more intensive livestock management, which includes frequent communal grazing, elevated herd densities, and limited access to clean water sources, can increase animal-to-animal contact and environmental contamination, thereby facilitating greater transmission potential. Conversely, the comparatively dispersed animal holdings, lower herd connectivity, and semi-arid climate of Lakki Marwat likely diminish both environmental oocyst survival and effective contact rates among hosts. Such conditions may reduce the sustained transmission cycles observed in Kohat. This interpretation aligns with prior studies highlighting the influence of local climatic conditions and husbandry practices on *Cryptosporidium* epidemiology in small ruminants.

Giadinis et al. [37] reported a notably higher prevalence of 76.6%, differing significantly from our results. Conversely, studies conducted by Sturdee et al. [38] aligned more closely with our findings, reporting a prevalence of 23.2%. Additional global comparisons revealed variable prevalence rates, with [39–41] reporting rates of 24%, 24.2%, and 20% in Romania, China, and Trinidad and Tobago, respectively. Our recorded prevalence rates in Bannu (20%), Lakki Marwat (18.33%), and Kohat (23.33%) echoed these global variations. However, some regions reported lower prevalence rates, such as Greece (7.1%), Belgium (9.5%), and Iran (17%). Notably, our study’s prevalence estimate of 20.55% was relatively higher than Greece and Belgium but comparable to Iran [31, 42, 43]. A study by Kabir et al. [44] found a much lower prevalence of 13.4% (2/15) in *C. hircus* from Turkey, which contrasts with the higher prevalence rates observed in our study. Similarly, Chikweto et al. [45] reported a prevalence of 24.4% in goats in Grenada, which is notably higher than our findings but still within the range of the global variation observed. In comparison, Shafieyan et al. [46] reported a lower prevalence of 6.18% in goats from Lorestan province, Iran, which further underscores the variability in prevalence rates across different geographical regions. Additionally, the overall prevalence of *Cryptosporidium* spp. in goats in Ecuador was reported at 18.2% by Celi et al. [47], which closely aligns with the prevalence observed in our study in District Lakki Marwat (18.33%). This finding suggests that, while regional differences exist, *Cryptosporidium* infection in goats is widespread, and its prevalence is subject to local factors such as environmental conditions, management practices, and geographical location.

Additionally, a Spanish study documented a wide range of 40–70% prevalence, contrasting with our infection rate estimated at 20.55%. These variations may be attributed to differences in hygienic conditions, study design, animal health, immunity, and management practices, highlighting the multifactorial nature of *Cryptosporidium* prevalence [30]. This study estimated an overall prevalence of *Cryptosporidium* infection at 20.55% among *C. hircus* across three distinct study areas. Notable district-wise variations were observed, with the highest prevalence in District Kohat (23.33%), followed by District Bannu (20%), and the lowest in District Lakki Marwat (18.33%). When compared to existing literature, marked differences in prevalence rates were noted across various regions. These findings are lower compared to the study by Rafiq et al. [1], who reported a significantly higher detection rate (61.00%–63.33%) in fecal samples collected from goats in the Mardan district, Pakistan. For instance, Giadinis et al. [37] reported a significantly higher prevalence of 76.6%, which contrasts sharply with our findings. Conversely, studies by Siddiki et al. [48] and Sturdee et al. [38] yielded prevalence rates of 15% and 23.2%, respectively, aligning more closely with our results. Global comparisons further illustrate variability in prevalence; reports from Romania, China, and Trinidad and Tobago documented rates of 24%, 24.2%, and 20%, respectively. Our findings in Bannu (20%), Lakki Marwat (18.33%), and Kohat (23.33%) resonate with these global patterns. Notably, other regions such as Greece (7.1%), Belgium (9.5%), and Iran (17%) exhibited lower prevalence rates. Our study’s prevalence of 20.55% is relatively higher than that of Greece and Belgium but comparable to Iran. These differences in prevalence rates can be attributed to several factors, including variations in hygienic conditions, study designs, animal health, immunity status, and management practices across regions. Moreover, the lack of statistical validation in comparing some of these studies may contribute to the apparent discrepancies, as differences in sample size, methodologies, and geographic factors can significantly influence outcomes. This emphasizes the multifactorial nature of *Cryptosporidium* prevalence and the necessity for context-specific investigations [49].

In dissecting the monthly prevalence of *Cryptosporidium* infection in *C. hircus*, descriptive monthly variation was observed, although differences among months were not statistically significant. August emerged as the month with the highest overall prevalence, estimated at 36.67%, followed by May (30%) and June, July, and October (26.67%). In contrast, January and February exhibited the lowest prevalence at 6.67%. Geographical disparities mirrored these temporal fluctuations, with District Kohat registering the highest prevalence (23.33%), followed by District Bannu (20%), while District Lakki Marwat reported the lowest prevalence (18.33%). The observed monthly variations may be attributed to environmental factors, specifically heavy rains and warm conditions conducive to oocyst survival, as noted by Perch et al. [50]. Warmer months, characterized by increased water intake from streams, ponds, and rivers, could contribute to elevated *Cryptosporidium* prevalence, a phenomenon emphasized by Sunderland et al. [51]. This exploration of monthly prevalence adds depth to our understanding of *Cryptosporidium* dynamics, shedding light on environmental influences that shape infection patterns among *C. hircus*.

Examining the seasonal prevalence dynamics of *Cryptosporidium* infection, our investigation identified distinctive patterns. The highest prevalence was documented during the summer season (30%), followed by spring and autumn (23.33% and 18.89%, respectively), while the winter season exhibited the lowest prevalence (10%). This observed seasonality aligns with findings of Perch et al. [50], underscoring a robust correlation between *Cryptosporidium* prevalence and climatic conditions. Our results strongly resonate with the notion that cryptosporidiosis exhibits pronounced seasonality, with peak prevalence coinciding with hot, warm, and rainy seasons of the year, as noted by Perch et al. [50]. The observed heightened prevalence during seasons characterized by heavy rainfall and elevated ambient temperatures aligns with the global distribution of cryptosporidiosis in wet and warm seasons, a phenomenon elucidated by Jafari et al. [52] and Green et al. [53]. This conformity with existing research underscores the impact of climatic variables on the prevalence dynamics of *Cryptosporidium* infection, further emphasizing the intricate interplay between environmental factors and parasitic occurrence.

Our investigation reveals a notable age-dependent trend in *Cryptosporidium* prevalence among *C. hircus*. The highest overall prevalence was documented at an early age, with the age group of ≤ 15 days exhibiting the highest percent prevalence (33.93%), followed by 16–30 days (17.69%), while the lowest prevalence was observed in the age group of 31–60 days (11.01%). Comparatively, our results indicate lower prevalence rates than those reported by Giadinis et al. [37], who observed a prevalence rate of 76.4% in diarrheic kids, and Delafosse et al. [54], who documented elevated prevalence in calves with diarrhea in a French study. Concordantly, studies by Siddiki et al. [48] in Bangladesh, Misic and Abe [55] in Serbia, and Harandi and Ardakani [42] in Iran collectively reinforce the vulnerability of young *C. hircus* and calves to *Cryptosporidium* infection. The heightened susceptibility of kids up to 3 months old, as highlighted by Siddiki et al. [48], could be attributed to their underdeveloped immune systems, rendering them more sensitive to infection. These findings underscore the significance of age as a contributing factor to *Cryptosporidium* prevalence dynamics, shedding light on the unique vulnerabilities of young animals to this parasitic infection.

Our investigation explores gender-specific variations in *Cryptosporidium* prevalence among *C. hircus*, revealing intriguing insights. The study reports a slightly elevated percent prevalence in female kids (22.22%) compared to their male counterparts (19.19%). This aligns with the findings of Siddiki et al. [48], who also noted a higher prevalence in female *C. hircus*. The observed gender disparity could be attributed to hormonal variations, as suggested by Danladi and Ugbomoiko [56]. Hormonal influences may contribute to differences in susceptibility to *Cryptosporidium* infection between male and female *C. hircus*. The nuanced interplay between hormonal factors and parasitic susceptibility warrants further investigation to comprehensively understand the mechanisms influencing gender-specific prevalence patterns. Our study adds valuable insights to the ongoing discourse on the role of gender in shaping *Cryptosporidium* infection dynamics among young goats.

This study has several limitations that should be considered when interpreting the findings. First, although the sample size was sufficient for preliminary insights, it may not fully capture the diversity of the broader goat kid population across the studied districts, which could limit the generalizability of the reported prevalence rates. Second, the diagnostic approach relied solely on modified Ziehl-Neelsen staining, a standard but imperfect method with a reported sensitivity of approximately 70–80% and specificity above 90%. This limitation likely leads to underestimation of true infection prevalence due to potential false negatives, especially in animals with low oocyst shedding, and precludes species-level identification of *Cryptosporidium*. Third, heterogeneity in animal management practices, environmental exposure, and health status among the goat populations across districts may have contributed to observed prevalence differences. Acknowledging these constraints is essential for proper contextualization of our results and highlights the need for future studies employing more sensitive molecular diagnostics and larger, more representative sampling to better elucidate the epidemiology of *Cryptosporidium* infections.

## 5. Conclusions

This study found a 20.55% prevalence of *Cryptosporidium* infection in *Capra hircus*, with the highest rate in District Kohat (23.33%). Infection rates were influenced by seasonal factors, particularly warm and rainy periods that favor oocyst survival. Younger kids (≤ 15 days) were more susceptible, likely due to immature immunity, and a slight gender difference hints at physiological influences on infection risk. These results highlight the zoonotic risk posed by *Cryptosporidium* in goat populations and stress the need for tailored control strategies. Recommended measures include improving hygiene in animal housing and communal grazing areas, ensuring access to clean water, educating farmers about risk periods and vulnerable animals, and implementing regular herd screening to monitor and control infection. By elucidating the epidemiological patterns and risk factors, this study provides essential insights to guide effective interventions and reduce public health impacts in southern Khyber Pakhtunkhwa and comparable regions.

## Data Availability

The datasets used and/or analyzed during the present study are available from the first author upon reasonable request.

## List of abbreviations

C.I., confidence interval; DNA, deoxyribonucleic acid; LGA, Local Government Area; MARA, Ministry of Agriculture and Rural Affairs; SPSS, Statistical Package for the Social Sciences; UVAS, University of Veterinary and Animal Sciences; °C, degree Celsius; gm, grams; h, hour; kg, kilogram; m, meter; min, minute; mm, millimeter; μm, micrometer; rpm, revolutions per minute.
